# Determinants of Cue-Elicited Alcohol Craving and Perceived Realism in Virtual Reality Environments among Patients with Alcohol Use Disorder

**DOI:** 10.3390/jcm10112241

**Published:** 2021-05-21

**Authors:** Olga Hernández-Serrano, Alexandra Ghiţă, Jolanda Fernández-Ruiz, Miquel Monràs, Antoni Gual, Mariano Gacto, Bruno Porras-García, Marta Ferrer-García, José Gutiérrez-Maldonado

**Affiliations:** 1 Department of Psychology, Catholic University of Saint Anthony, Av. de los Jerónimos, 135, Guadalupe de Maciascoque, 30107 Murcia, Spain; ohernandez@ucam.edu; 2 Department of Psychology, Health and Technology, University of Twente, Drienerlolaan, 5, 7522 NB Enschede, The Netherlands; alexandra.ghita@utwente.nl; 3 Department of Clinical Psychology and Psychobiology, Faculty of Psychology, University of Barcelona, Passeig de Vall d’Hebron 175, 08035 Barcelona, Spain; jolandafruiz@gmail.com (J.F.-R.); brporras@ub.edu (B.P.-G.); martaferrerg@ub.edu (M.F.-G.); 4 Addictive Behaviors Unit, Hospital Clinic of Barcelona, Carrer de Villarroel, 170, 08036 Barcelona, Spain; mmonras@clinic.cat (M.M.); tgual@clinic.cat (A.G.); 5 Department of Physical Therapy, Faculty of Medicine, Campus Espinardo, University of Murcia, 30100 Murcia, Spain; marianogacto@um.es

**Keywords:** alcohol use disorder, alcohol craving, virtual reality, cue exposure, perceived realism

## Abstract

The identification of variables that can modulate the efficacy of cue exposure using virtual reality (VR) is crucial. This study aimed to explore determinant variables of cue-elicited alcohol craving and perceived realism (PR) of environments and alcoholic beverages during a VR cue-exposure session among alcohol use disorder (AUD) outpatients. A prospective cohort study was conducted amongst 72 outpatients with AUD from a clinical setting. Alcohol craving experienced during VR exposure and PR of virtual environments and alcoholic drinks were evaluated after a VR session of exposure to alcohol-related contexts and cues. Sociodemographic, psychological and consumption characteristics were examined as possible predicting variables. Multiple linear regression analyses showed that the AUD severity and PR of beverages were predictors of cue-elicited alcohol craving. Educational level, PR of beverages and age were predictors of the PR of VR environments. In relation to the PR of VR beverages, cue-elicited alcohol craving and the PR of environments were predictors. A simple mediational model was also performed to analyze the influence of the PR of beverages on the relationship between the AUD severity and alcohol craving experienced during VR exposure: an indirect or mediational effect was found. PR of alcoholic beverages was (1) a key predictor of the PR of VR environments (and vice versa) and the alcohol craving (and vice versa) experienced during VR cue-exposure sessions using ALCO-VR software among AUD patients and (2) a mediator between AUD severity and cue-elicited alcohol craving.

## 1. Introduction

Drug craving is one of the prominent mechanisms in the spectrum of substance use disorders (SUDs), with long-term clinical implications in the development of the psychopathology, course of treatment and abstinence maintenance [1,2,3]. Craving is understood as an impetuous urge to consume the substance [4,5,6], which may result in compulsive substance-seeking and use-related behaviors [7,8]. Alcohol craving is a subjective perception of drinking-related desire, a “wanting” continuum [9], which prompts psychophysiological [10], emotional [11], cognitive [12] and behavioral [13] responses in individuals with alcohol use disorder (AUD). To articulate its clinical salience [5], alcohol craving has been targeted in assessment [14,15,16] and psychological treatment approaches [17,18,19], in addition to relapse prevention interventions [20].

One of the most extensively used techniques in exploring craving relies on the commonly known paradigm of cue exposure (CE), which implies systematic in vivo, imagery or multimedia exposure to alcohol-related stimuli [21,22,23]. The theoretical framework of CE relies on the principles of classical conditioning [24]. Over time, alcohol-related cues and contexts acquire incentive motivational properties expressed in alcohol craving, which may further result in drinking-related behaviors [9,25]. To gain more insights into the underlying mechanisms of AUD, the CE method has been consistently implemented to induce elicitation of alcohol craving [26,27,28] or to gradually reduce craving as part of a comprehensive psychological treatment (e.g., systematic desensitization therapy) [29,30,31]. Systematic desensitization emphasizes the CE paradigm that involves repetitive exposure to alcohol-related stimuli without performing any drinking-related behaviors [23]. That is, the individual is exposed to alcoholic beverages, and the therapeutic protocol consists of disclosing cognitive–emotional–physiological and behavioral responses to alcohol stimuli with the aim to develop coping skills to be further implemented in real-life settings [26]. Such exposure and response prevention aims at behavioral modifications of the AUD individuals [23,26,29]. Over time, the exposure process may lead to habituation to alcohol stimuli, and the goal of the CE technique is to gradually reduce alcohol craving by extinguishing initially conditioned responses to alcohol stimuli [23,32]. Regardless of the extensive body of literature using the CE paradigm, two meta-analyses emphasized the inconsistency of results regarding the effectiveness of this technique in the field of SUD, particularly due to the simplistic and limited stimuli inputs, which limits therapy effects transferred into real-life settings of the AUD individuals [23,33].

Nevertheless, the CE paradigm may benefit from more sophisticated technologies like virtual reality (VR) [1,34,35]. VR provides 3D simulations of real situations through multiple sensory inputs like auditory, olfactory, visual or tactile inputs [36]. Such a comprehensive approach provides a better alternative to traditional CE by inducing the individual’s sense of presence, a subjective state-of-mind of experiencing virtual environments similar to naturalistic contexts [37,38,39]. This is particularly due to VR technical features such as immersion within the VR environments and interaction with the system through real-time feedback [40], as well as including significant substance-related stimuli and environments with a moderate to high degree of realism [41,42,43]. Perceived realism (PR) refers to the subjective perception of the individual regarding the fidelity of the VR environment (does the virtual environment recreate the realistic environment?) [44]. Related to psychology, VR provides sensory-based information to enhance individual experiences similar to naturalistic environments. Therefore, VR has proven its ecological validity in the field of clinical psychology as an assessment and treatment method of different mental health disorders [45].

According to previous research, there are several concurrent predicting variables involved in cue-elicited substance craving, namely sociodemographic factors and clinical correlates. In terms of sociodemographic factors, age is an important predictor, suggesting that younger individuals experience greater craving responses compared to older SUD participants [46]. In terms of gender, women tend to experience higher levels of cue-induced craving compared to men [47]. However, other studies found no relationship between gender and craving as triggered by the CE paradigm [48,49]. Moreover, another study found that individual variables such as age, gender, education or employment were not significant predictors of cue-induced craving [50]. Nevertheless, other possible predicting variables such as socioeconomic or civil status were less explored in relationship with cue-induced craving in CE paradigms.

Regarding clinical correlates, there are multiple factors associated with cue-elicited craving. For instance, higher levels of stress-induced craving predict relapse in AUD individuals [51]. The strong association of alcohol craving in relation to AUD symptoms has been highlighted [52]. Such studies show the interplay between alcohol craving and AUD severity. Furthermore, neuro-psycho-physiological responses are a robust predictor of abstinence duration in the following months post-treatment [53,54], but there is less empirical evidence regarding the abstinence period as a predicting variable of cue-induced alcohol craving. In addition, the presence of psychiatric comorbidities such as chronic stress and anxiety [54], depression [55], personality disorders [56], eating disorders [57] and concurrent use of illicit drugs [58] impacts the AUD treatment course and recovery. Another fundamental aspect related to CE paradigms is attentional bias [35], which refers to the implicit processing of substance-related cues while neglecting other stimuli [59]. Accordingly, attention to substance stimuli arouses psychophysiological reactivity in individuals with problematic use of substances, which is an essential mechanism of the CE effectiveness.

In VR-CE paradigms, a recent study has found that the sense of presence augments alcohol craving more significantly in individuals with a heavy drinking pattern compared to individuals with occasional drinking patterns, indicating that the severity of substance misuse and perceived ecological validity of the VR environment are important variables in terms of craving elicitation [60]. Another study determined that sense of presence can predict craving during exposure to VR environments [61]. An additional study indicated that how substance-related cues are perceived by the individual could induce expectations regarding the availability of the substance in the environment, which, in turn, can trigger craving. [59]. Although some authors found that participants with AUD experienced high levels of presence and experienced emotions in VR congruent with their emotions in the real world [41], other authors, in contrast, did not find this congruence between the perception of reality and the emotion felt in the virtual environment [43]. Finally, research on VR suggests that there is a strong and synergistic association between the sense of presence in the virtual environments and the emotional reactivity (such as the anxiety) of users in these environments [62,63].

Few studies have examined what variables could predict cue-elicited alcohol craving responses among AUD patients, and, in some cases, these studies did not provide a consistent pattern of results. In relation to PR in virtual environments, these studies have focused on (1) evaluating only the average rating of “realism/presence” among individuals with AUD [41] and cocaine-dependent subjects [43], (2) testing whether the realism is a mediator or a moderator of the relationship between alcohol history and postimmersion alcohol craving [60] and (3) studying the relationship between sense of presence and emotions [63,64]. Consequently, there is a lack of literature reporting on variables that could predict PR in virtual environments and alcoholic drinks. Given this knowledge gap, this study aimed (1) to explore sociodemographic (age, gender, education, socioeconomic and civil status) and psychological and consumption variables (AUD severity, abstinence time, psychiatric comorbidity, trait anxiety, attentional bias, tobacco use, illicit substance use, alcohol craving experienced during the previous week) as predictors of cue-elicited alcohol craving (“intensity of alcohol craving experienced during VR exposure”), PR of virtual environments, and PR of alcoholic beverages during a VR cue-exposure session among AUD outpatients; (2) to evaluate if the PR of virtual environments is a predictor of cue-elicited alcohol craving (“intensity of alcohol craving experienced during VR exposure”) and PR of alcohol beverages; (3) to explore the PR of alcoholic beverages as a predictor of cue-elicited alcohol craving (“intensity of alcohol craving experienced during VR exposure”) and the PR of virtual environments; (4) to analyze alcohol craving (“intensity of alcohol craving experienced during VR exposure”) as a predictor of PR of virtual environments and PR of alcoholic beverages; and (5) to seek to identify, through a simple mediational model, the mechanism underlying the relationship between AUD severity and alcohol craving (“intensity of alcohol craving experienced during VR exposure”) via the inclusion of PR of alcoholic beverages as the mediator variable.

## 2. Materials and Methods

### 2.1. Participants

A total of 95 participants were identified during the study period. Of these, 16 either did not fulfill the inclusion criteria or were excluded for other reasons (e.g., uncorrected visual impairment or work commitments clashing with the treatment schedules). The inclusion criteria were an AUD diagnosis according to the criteria of the Diagnostic and Statistical Manual of Mental Disorders (5th Ed.) (American Psychological Association, APA, 2013) [65] and receiving treatment-as-usual (TAU) for AUD at the Hospital Clinic of Barcelona. The lead clinical psychologist from the hospital selected these patients based on their clinical history, while the patients were under ambulatory treatment (receiving TAU) at the moment of this study. This outpatient treatment includes pharmacotherapy and psychosocial care. The exclusion criteria were severe psychopathology (e.g., psychosis), severe cognitive impairment, use of anti-craving medication (e.g., naltrexone) and pregnancy. A total of 79 AUD patients, aged 36 to 67 years, were enrolled in the study. Of these, 7 participants voluntarily dropped out from the study, and their data were excluded as they did not complete all the assessment tests. Finally, 72 participants completed all the assessment phases of the present study. The ethical code number is 0377 (HCB/2017/0377) and the approval date was 09/2017.

### 2.2. Measures

Alcohol Use Disorder Identification Test (AUDIT) [66] was used to screen alcohol consumption, drinking behaviors and problematic alcohol-drinking patterns. The AUDIT is a 10-item screening questionnaire aiming to detect harmful alcohol consumption, dependence and consequences of continued drinking. The items score from 0 to 4 points, except questions 9 and 10, scoring 0, 2 or 4 points. The final score ranges from 0 to 40 points. A final score equal to or higher than 8 points is the cut-off score to indicate hazardous drinking and warrants further assessment for possible AUD [66,67]. In our study, AUDIT was also used as a severity indicator of AUD, as seen in previous research [67].

Multidimensional Alcohol Craving Scale (MACS) [68] was used in the study to explore the “intensity of alcohol craving experienced by the participant in his/her previous week”. MACS has 12 items with scores ranging from 1 (“strongly disagree”) to 5 (“strongly agree”). There are three MACS outcomes: “desire to drink”, “behavioral disinhibition” and “global score”, scored as non-existent (0–12), mild (13–22), moderate (23–40) or intense (>40) craving.

Multidimensional Alcohol Craving Scale—Virtual Reality (MACS-VR) was implemented in the protocol as an ad hoc modified version of MACS. The items, scores and outcomes of MACS-VR remained the same as in the MACS original version, including the “desire to drink” and “behavioral disinhibition” subscales. In contrast to MACS, the aim of the MACS-VR was to determine the “intensity of alcohol craving experienced during VR exposure”.

Spanish Alcohol Stroop Task [69] was included in the study to explore the interference effect for alcohol content. The task consists of three parts, with alcohol-related words in black–white and color. Over 45 s, the participant is instructed to read the words as fast as possible. Final scores of the Stroop task indicate attentional bias towards alcohol content.

State-Trait Anxiety Inventory (STAI) (the trait part) [70] was used to determine trait anxiety. The subscale of the STAI consists of 20 items aiming to depict trait anxiety. All items are rated on a 4-point scale (0 = ‘‘not at all’’ to 3 = ‘‘very much so’’). Higher scores indicate greater anxiety.

Self-reported perceived realism was included in the study as an ad hoc scale to determine the subjective perception of VR alcohol cues and contexts. The two-item scale (“On a scale from 0 to 10, 0 being poor realism and 10 being excellent realism, how do you rate the virtual alcoholic beverages?” and “On a scale from 0 to 10, 0 being poor realism and 10 being excellent realism, how do you rate the virtual environments?”) focused on the perceived realism of the *alcoholic beverage* and *alcohol-related environments* within the VR system as in previous research [24]. Scores of each item ranged from 0 (“poor VR realism”) to 10 (“excellent VR realism”).

### 2.3. Instruments

#### 2.3.1. Hardware

The VR equipment consisted of an Oculus Rift S head-mounted display (HMD), 1080 × 1200 resolution per eye, a 90 Hz refresh rate, and 110o field of view, sensors, touch controllers and a computer compatible with VR technology (INTEL^®^ Core™ i7-2600 CPU, 16.0 GB RAM, Operating System 64 bits, processor × 64, and a graphics card NVIDIA GeForce GTX 1080 Ti).

#### 2.3.2. Software

**Figure 1:**
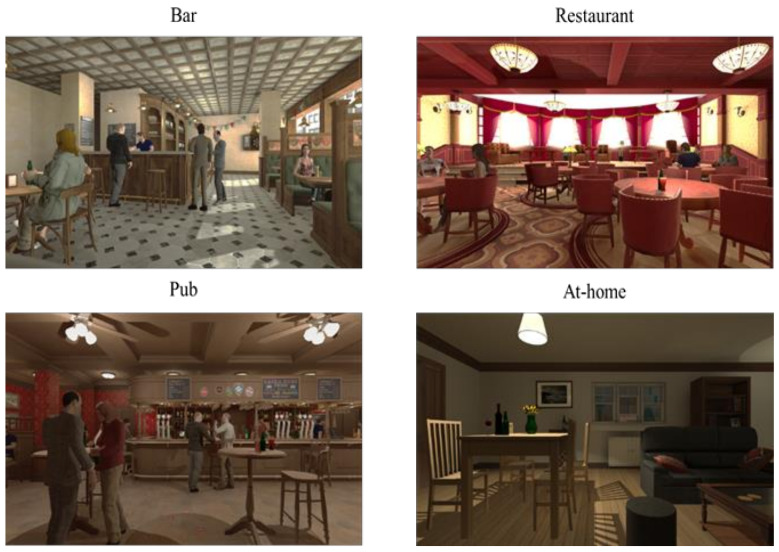
Images of the “ALCO-VR” software.

The current study is part of a broader project. The software used, “ALCO-VR” (developed by the VR-Psy Lab, University of Barcelona, Barcelona, Spain), was created considering the outcomes of a previous study, in which we determined triggering factors of alcohol craving in AUD individuals [71]. Variables such as VR alcohol-related environments (Figure 1, pub, bar, restaurant and at-home environment), social interaction (VR environments with and without human avatars), VR alcohol cues (e.g., menu of 22 alcoholic drinks; backgrounds with alcohol bottles) or different times of day (daytime or nighttime) were associated with greater levels of alcohol craving in AUD patients. Hence, the “ALCO-VR” platform simulated naturalistic contexts considering patients’ experiences and included two environments during daylight (bar and restaurant) and two environments during nighttime (pub and at-home), one environment with no social interaction (the at-home environment) and three environments with social interaction (bar, pub, restaurant). To approximate the VR experience to realistic contexts, the users could approach their preferred drink and observe it from all angles using real-time movement feedback with Oculus Touch controllers. In this way, a high level of interaction between the platform and the user enhanced the sense of presence within the VR environments. A previous study determined that the “ALCO-VR” software induced greater levels of cue-induced craving in AUD patients compared to social drinkers [24].

The software consists of two parts (assessment and therapy), but in regards to the current study, solely the assessment part is further presented. Prior to the VR alcohol-related experience, the software includes a neutral environment, which consists of a white room and a glass of water. This neutral environment is fundamental to familiarize users with the VR technology. The system allows the selection of preferred alcoholic beverages and creates a hierarchy based on users’ self-ratings of alcoholic drinks and environments. Hence, the assessment part consists of exposure to VR alcohol content from the lowest-rated environment with the lowest-rated alcoholic drink to the highest-rated environment and the highest-rated alcoholic drink.

### 2.4. Procedure

The study was approved by the ethics committees from the University of Barcelona and Hospital Clinic of Barcelona. Patients were recruited from the Addictive Behaviors Unit, Hospital Clinic of Barcelona, and were included in the study based on their informed written consent. The single-session appointment consisted of collecting sociodemographic and anamnesis data from the AUD patients, including medication, abstinence and use of substances other than alcohol. Urine analyses were performed to confirm self-reports of the patients regarding their substance use. In addition, patients were asked to complete the AUDIT, MACS (“intensity of alcohol craving experienced during the previous week”), STAI (trait subscale) and Stroop. Then, participants were given instructions on how to use the VR equipment and became familiar with the VR technology upon completion of the neutral environment. They were asked to approach each drink within the VR environments and observe it from all angles without attempting to drink.

**Figure 2:**
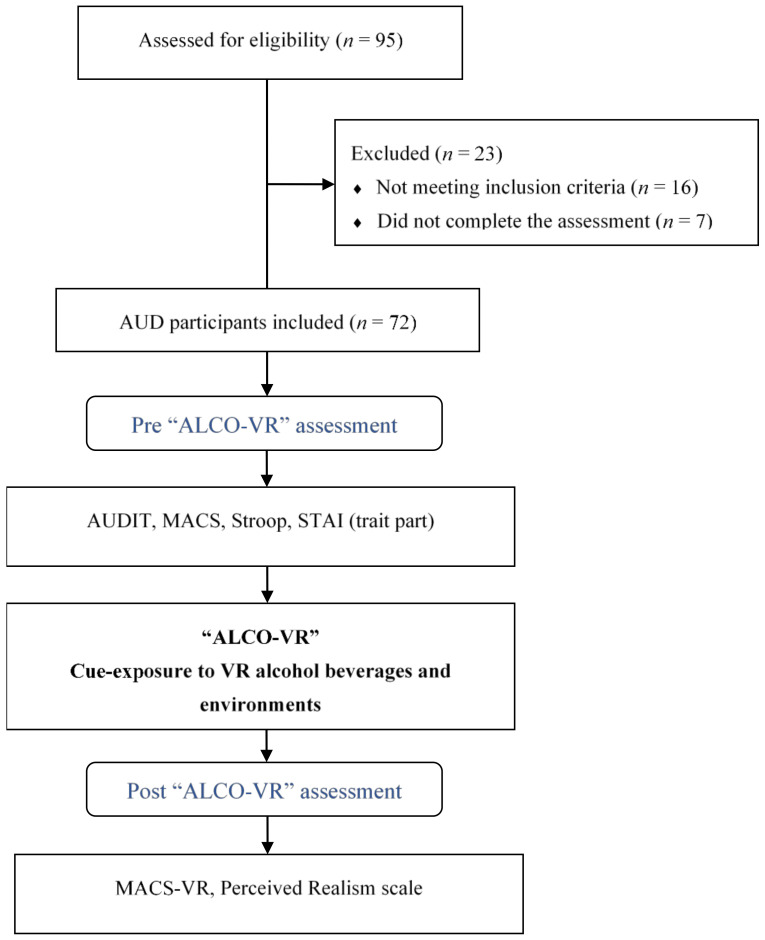
Flowchart of the study. AUD = alcohol use disorder; AUDIT = Alcohol Use Disorder Identification Test; MACS = multidimensional alcohol craving scale; MACS-VR = multidimensional alcohol craving scale-virtual reality; STAI = trait- anxiety inventory; VR = virtual reality.

Exposure to VR alcohol environments gradually increased from the lowest-rated drink and the lowest-rated VR environment to the highest-rated alcoholic beverage and highest-rated environment. To enhance the VR experience, olfactory stimuli were introduced during the exposure procedure. Alcoholic drinks were transferred on cotton pads and placed next to the participants, corresponding to the beverages exhibited within the VR environments. The VR experience lasted for a maximum of 15 min. Following this part, patients were asked to complete the MACS-VR (“intensity of alcohol craving experienced during VR exposure”) and the self-reported perceived realism scale (see Figure 2). The VR cue-exposure session was delivered by experienced therapists at the VR-Psy Lab of the University of Barcelona.

Lastly, the therapists performed the debriefing interview. After the session, patients received short debriefings with the aim of disclosing any cognitive, emotional and behavioral correlates of future alcohol use. The ultimate goal of the debriefing was to minimize craving and any further alcohol consumption. None of the participants left the laboratory setting until they reported an alcohol craving rating between 0 and 2 points on a 0-to-10-point scale.

### 2.5. Statistical Analysis

Descriptive statistics were used to characterize the cohort at baseline, including sociodemographic, psychological and consumption characteristics. The arithmetic mean and standard deviation of continuous variables and the percentage of counting variables were calculated.

Next, associations were tested for a relationship between sociodemographic, psychological and consumption variables (age, gender, education, socioeconomic and civil status, AUD severity, abstinence time, psychiatric comorbidity, trait anxiety, attentional bias, tobacco use, illicit substance use, intensity of alcohol craving experienced during the previous week), and the scores in the following quantitative variables: (1) the intensity of alcohol craving during VR exposure, (2) PR of the virtual environments and (3) PR of the alcoholic beverages. Independent *t*-test and analyses of variance (ANOVA) were used for categorical variables (with two or more than two categories), and Pearson correlation was used for quantitative variables.

Moreover, the PR of environments and PR of alcohol beverages were analyzed to determine whether there was a relationship with the alcohol craving experienced during VR exposure and, at the same time, to determine the correlation between them (PR of environments in the PR of alcoholic beverages). Pearson correlation was used for these analyses.

Solely the variables reaching statistical significance at the *p* < 0.05 level in the previous analyses were included in the multiple regression analysis with intensity of alcohol craving experienced during VR exposure, PR of environments and PR of alcoholic beverages as the dependent variables. The final model was produced by the enter method. Analyses were performed using the statistical package SPSS version 24.0 (IBM Corp., Armonk, NY, USA).

Finally, a simple mediational analysis was also conducted to assess the effect of PR of alcoholic beverages on the relationship between AUD severity and alcohol craving (“intensity of alcohol craving experienced during VR exposure”). Indirect effects were tested using the software PROCESS [72] and using an empirical bias-corrected bootstrapping procedure with 10,000 resamples and a 95% confidence interval.

## 3. Results

Participants’ characteristics at baseline are described in Table 1. A total of 72 AUD patients (55.6% males) with a mean age of 52.17 years (*SD* = 8.83) participated in the VR cue-exposure session. University education was present among 41.7% of participants, whilst a high percentage of patients had medium socioeconomic status (80.6%). As for the civil status, most patients were married/in a relationship (45.8%) or separated/divorced (29.2%). Almost half of the sample (45.8%) reported psychiatric comorbidities. At baseline, the mean time of abstinence was approximately three months, whereas the average scores in AUDIT, alcohol craving during the previous week and trait anxiety were 15.94, 27.8 and 27.1, respectively. Finally, the mean score of PR of the environments was higher than the mean of PR of beverages (7.90 versus 6.93).

The results on the relationship between sociodemographic, psychological and consumption variables (at baseline) with MACS-VR (alcohol craving experienced during VR exposure), PR of environments and PR of beverages (at post-test) are presented in Table 2. No statistical differences were found with the independent t-test; however, we found significant differences with the analysis of variance (ANOVA) and the Pearson correlation. Differences were found in the different types of civil status in relation to alcohol craving experienced during VR exposure and in the different levels of education and PR of environments. On the other hand, an inverse and significant correlation was found between age and alcohol craving experienced during VR exposure; and direct and significant correlations existed (1) between AUD severity and trait anxiety with alcohol craving experienced during VR exposure, (2) between age and PR of environments and (3) between AUD severity and PR of alcoholic beverages.

Table 3 shows the results of the Pearson correlation in relation to the variables: PR of environments, PR of beverages and alcohol craving experienced during VR exposure. Direct and significant relationships were found (1) between PR of beverages and alcohol craving experienced during the VR exposure and (2) between PR of beverages and PR of environments.

Table 4 presents the results from the multiple regression analysis. A multiple regression was run to predict the intensity of alcohol craving during VR exposure from civil status, age, AUD severity, trait anxiety and PR of alcoholic beverages. The multiple regression model statistically significantly predicted alcohol craving levels, *F* = 5.926, *p* < 0.001, accounting for 31% of the explained variability: AUD severity and PR of beverages made a statistically significant unique contribution to the model, *p* < 0.05. As for the PR of the environments, three variables were significant (*p* < 0.05): education level, PR of alcoholic beverages and age. The coefficients were −0.336, 0.444 and 0.036, respectively (*F* = 17.708, *p* < 0.00, *R*^2^ = 0.439). On another note, AUD severity, PR of environments and alcohol craving during VR exposure (*p* < 0.05) significantly predicted PR of beverages (*F* = 16.715, *p* < 0.00, *R*^2^ = 0.424). Regression coefficients and standard errors can be found in Table 3.

**Figure 3:**
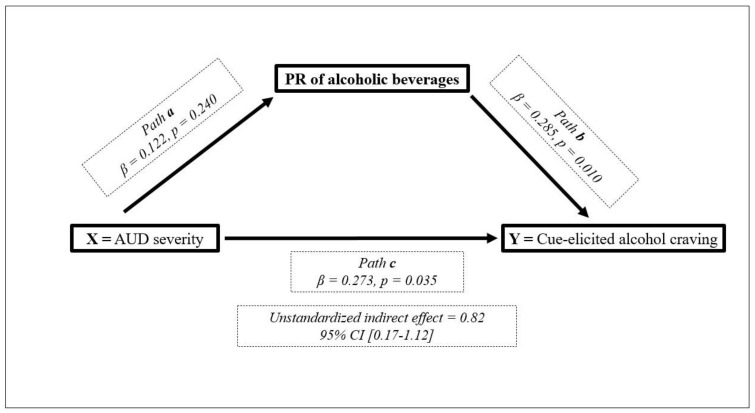
Mediational model.

As for the mediational effect of the PR of alcoholic beverages on the relationship between AUD severity and cue-elicited alcohol craving, the results of the simple mediation analysis revealed that the PR of alcoholic beverages was a mediator of this relationship, since all the postulates were met except for path a, “AUDIT predicts PR”, in which the results were not statistically significant (*p* = 0.240). There was, nonetheless, an indirect effect, since the bootstrapped unstandardized indirect effect was 0.82, and the 95% confidence interval ranged from 0.17 to 1.12. The indirect effect was therefore statistically significant (see Figure 3).

## 4. Discussion

The first objective of this study was to examine whether sociodemographic, psychological and consumption variables would predict the scores of intensity of alcohol craving experienced during VR exposure, PR of environments and PR of alcoholic beverages during a VR cue-exposure session in outpatients diagnosed with AUD. In accord with previous studies among AUD patients [73,74], AUD severity is a predictor of cue-elicited alcohol craving in our study. Concerning previous VR studies exploring alcohol cue reactivity, some authors found a strong correlation between AUD severity and alcohol craving using different assessment instruments [24]. This association was also present in studies on tobacco users [75] and alcohol and tobacco co-users [76].

The results of the present study show that the PR of alcoholic beverages is a predictor of cue-elicited alcohol craving and vice versa. Three possible and different explanations could justify this finding. First, previous studies found a synergistic relationship between high emotions (e.g., anxiety) and a high sense of presence, including the dimension of realism, in therapeutic virtual environments [63,64]. In this sense, an AUD patient could subsequently interpret his/her emotions as evidence of the reality of his/her experience. Second, our perception of stimuli (e.g., VR environments and cues) may be augmented as a result of significant disorder-related information (for instance, olfactory inputs). According to our results, it is important to highlight that the patients received olfactory stimuli associated with the alcoholic beverage stimuli displayed at that time. For example, if they visualized a glass of beer in their hand, they immediately received the beer-scented stimulus. In this sense, a meta-analysis of cue-reactivity in addiction research found that the exposure to the smell of alcoholic beverages evoked increases in alcohol craving [77]. On the other hand, the relationship between olfactory stimuli and experienced realism (subjective realism) during VR-assisted exposure therapy has recently been explored [78]: the results showed that olfactory stimuli positively influenced the experienced realism when administered and that the realism decreased when olfactory stimuli were withheld [78]. Based on these observations, olfactory stimuli may be associated with higher levels of alcohol craving and the PR of alcoholic beverages. Third, according to the incentive sensitization theory, over time alcohol-related cues in individuals with alcohol misuse and AUD become highly sensitive and salient stimuli, which in turn draw automatic, implicit attention to processing salient information (target cue—glass of beer) while neglecting other environmental cues (VR environments). Further research on the relationship between alcohol craving experienced during VR exposure and the PR of alcoholic beverages is required.

Similar to the relationship between cue-elicited alcohol craving and the PR of beverages, we found that the PR of alcoholic beverages was a predictor of the PR of environments and vice versa (PR of environments was a predictor of the PR of beverages). In this sense, our results highlight the importance of the realism of alcoholic beverages in virtual reality programs to predict changes in the alcohol craving experienced during VR exposure and the PR of environments. However, no correlation was found between the intensity of alcohol craving experienced during VR exposure and the PR of environments. A possible explanation is that proximal cues (for instance, a glass of beer) are una-voidable for drinking to occur and, therefore, they coincide, all the time, with drinking virtually, whilst contextual cues (such as a restaurant) occur with drinking solely sometimes. For instance: if an individual always drinks in restaurants, that individual probably drinks, as well, in many other environments aside from restaurants, but he/she will never drink without the proximal cue of an alcoholic beverage. The association between drinking and environments may be therefore somehow mitigated by the relative number of different environments encountered over the course of drinking. This is supported by a study showing that smokers’ reactivity to environments (e.g., bar) was robust, although significantly less than that elicited by a set of proximal stimuli (e.g., lit cigarettes) [79]. Further research is required on the PR of alcoholic beverages.

An indirect or mediational effect of the PR of alcoholic beverages on the relationship between AUD severity and cue-elicited alcohol craving was found [60,61,80,81,82]. The impact of the PR of alcoholic beverages as a mediator is an explanation of how virtual reality can shape the behavior of participants. According to the mediation model, the AUD severity on reported cue-elicited alcohol craving is mediated by the perceived realism of alcoholic beverages. The AUD severity significantly affects the PR of beverages in an alcohol VR environment, which in turn affects the alcohol craving experienced during the VR exposure. A recent study tested whether the sense of presence was a mediator or a moderator of the relationship between past alcoholic experiences (i.e., being heavy or occasional drinker) and postimmersion alcohol craving [60]. The authors found a moderator effect of the perceived ecological validity of the environment on the relationship between alcohol drinker groups and self-reported craving. However, they did not find a mediator effect because several of the basic assumptions of the mediation model were not met. The differences between the study by Simon et al. [60] and the present study could be explained by the differences in participant type (nonclinical populations versus abstinent alcohol-dependent patients) or the type of dimension of the sense of presence (perceived ecological). Future studies should delve into exploring these characteristics and others as the specific aspects of alcoholic beverages (such as the iconicity or simplicity of the image) and their eventual impact on this relationship.

The age of AUD patients and the level of education were shown as predictors of PR of environments in the current study. This result may be explained by the fact that older adults and individuals with low educational level are less familiar with new technologies in their daily lives [83]. The cue exposure through VR may result in high scores of PR of environments. Furthermore, VR is nowadays being integrated into many different areas of our lives, especially among younger people, in a wide range from video games to educational and training tools for students. Technology has emerged as a useful tool in different university studies: its management becomes crucial to acquire knowledge and practical skills, as well as to enhance the quality of the academic process and develop new trends in teaching innovation [84]. Another feasible explanation of the relationship between the age of the patients and the perceptual realism of virtual environments could be associated with specific environments in which the patient develops social interaction. This idea is supported by several authors suggesting that older adults’ virtual experiences seem to differ from younger adults’ perceptions of virtual environments, underlining the importance of social interactions using avatars to experience higher levels of PR in virtual reality [85]. In the present study, VR environments were created considering social interactions, therefore including avatars in the environments. Hence, one environment (the at-home environment) had no social interaction, whilst three scenarios (bar, pub and restaurant) involved social interaction. Furthermore, the virtual environments were designed based on the daily experiences of the patients and the literature review. And we focused on variables such as presence of others, situations, time of the day, day of the week, mood and type of alcoholic beverage [71].

Our findings should be interpreted in light of our study’s methodological limitations. First, in relation to the term “PR of virtual environments” used in the present study, there seems to be no consensus on this concept. Differences in the use of the term are reflected in several studies. In some studies, it is defined as “perceived ecological validity of the environment” or “experienced realism” or “realism”, and these concepts are dimensions or subscales of the sense of presence. In other investigations, the term “realism” is identified as a dimension of physical presence. Moreover, other researchers use the terms “realism sensation”, “naturalness” or “realism/presence”. On the other hand, the sense of presence is a multidimensional concept including realism in its definition. However, only a few studies analyzed all dimensions independently. This lack of consensus about the concept “PR of environments” and the dimensionality of the sense of presence complicated the comparisons with other scientific studies on the same subject matter. Second, different types of instruments were used to measure alcohol craving (e.g., Visual Analogue Scale or Obsessive Compulsive Drinking Scale) and realism (e.g., ITC-Sense of Presence Inventory, iGroup Presence Questionnaire or Visual Analogue Scale) in the scientific literature, and this fact hinders the comparability and hence the discussion of the results. Third, in the present study, the PR of environments and PR of beverages were evaluated, but we did not evaluate other aspects that may influence the results, such as the realism of virtual characters. Recently, a study found that realism was a positive option for virtual characters in virtual reality environments [86]. Future studies should explore whether the PR of character appearance would predict high alcohol craving scores using ALCO-VR software.

## 5. Conclusions

In summary, the level of education and age were predictors of the PR of environments, and severity of AUD predicted alcohol craving experienced during VR exposure. Furthermore, PR of alcoholic beverages was (1) a predictor of the PR of VR environments (and vice versa: PR of environments was a predictor of PR of beverages) and the alcohol craving experienced during VR cue-exposure sessions (and vice versa: alcohol craving was a predictor of PR of beverages) using ALCO-VR software among AUD patients and (2) a mediator between AUD severity and cue-elicited alcohol craving.

## Figures and Tables

**Table 1:** Participant characteristics at baseline (*n* = 72).

Characteristics	Total Sample
	*N* (%)
Gender	
Female	32 (44.4%)
Male	40 (55.6%)
Psychiatric comorbidity (presence)	
Yes	33 (45.8%)
No	39 (54.2%)
Current smoker	
Yes	35 (48.6%)
No	34 (47.2%)
Illicit drug use in the month prior to baseline	
Yes	21 (29.2%)
No	50 (69.4%)
Education	
Elementary	0 (0.0%)
High school	25 (34.7%)
Junior college associate degree	12 (16.7%)
University	30 (41.7%)
Master’s degree	4 (5.6%)
Doctoral studies	1 (1.4%)
Socioeconomic status	
Low	11 (15.3%)
Medium	58 (80.6%)
High	2 (2.8%)
Civil status	
Single	11 (15.3%)
Married/in a relationship	33 (45.8%)
Separated/divorced	21 (29.2%)
Widower	6 (8.3%)
	***M* (*SD*)**
Age	52.17 (8.83)
AUDIT	15.94 (10.28)
Abstinence duration (days)	90.83 (110.77)
STAI-trait	27.13 (11.85)
Stroop Task	33.96 (155.92)
MACS	24.15 (7.90)

*M* = mean; *SD* = standard deviation; AUDIT = Alcohol Use Disorder Identification Test; MACS = Multidimensional Alcohol Craving Scale; STAI-trait = State-Trait Anxiety Inventory.

**Table 2:** The relationship between sociodemographic, psychological and consumption characteristics with MACS-VR, PR of environments and PR of beverages.

Characteristics	MACS-VR		PR of Environments		PR of Beverages	
	*M* (*SD*)	*t* (*p*)	*M* (*SD*)	*t* (*p*)	*M* (*SD*)	*t* (*p*)
Gender		−1.552 (0.125)		−0.257 (0.798)		−0.919 (0.361)
Female	30.50 (13.86)		7.96 (1.78)		7.18 (2.45)	
Male	25.77 (11.95)		7.86 (1.70)		6.72 (1.81)	
Psychiatric comorbidity		−0.564 (0.575)		−1.098 (0.276)		−0.635 (0.528)
Yes	23.39 (9.84)		7.66 (1.89)		6.75 (2.27)	
No	24.79 (11.02)		8.11 (1.57)		7.07 (1.99)	
Current smoker		0.235 (0.815)		0.860 (393)		1.316 (0.193)
Yes	27.91 (14.05)		8.08 (1.68)		7.20 (2.36)	
No	27.17 (11.96)		7.72 (1.83)		6.52 (1.82)	
Illicit drug use in the month prior to baseline		3.340 (0.831)		998. (321)		−0.357 (0.722)
Yes	35.42 (12.50)		7.16 (1.91)		6.76 (2.27)	
No	24.86 (12.03)		8.22 (1.58)		6.96 (2.06)	
	***M* (*SD*)**	***F* (*p*)**	***M* (*SD*)**	***F* (*p*)**	***M* (*SD*)**	***F* (*p*)**
Education		0.997 (0.427)		3.406 (0.008) *		1.837 (0.118)
Elementary	24.30 (12.34)		8.60 (1.34)		6.30 (2.31)	
High School	33.13 (15.72)		8.77 (1.48)		7.86 (1.64)	
Junior college associate degree	30.41 (14.58)		7.58 (1.16)		7.16 (1.33)	
University	25.30 (11.07)		7.58 (1.89)		6.86 (2.30)	
Master’s degree	29.50 (11.03)		7.75 (0.95)		5.75 (2.62)	
Doctoral studies	25.00 (NA)		3.00 (NA)		3.00 (NA)	
Socioeconomic status		0.318 (0.729)		1.295 (0.281)		1.337 (0.270)
Low	30.63 (14.65)		8.09 (1.22)		7.00 (2.19)	
Medium	27.60 (12.94)		7.95 (1.81)		6.98 (2.10)	
High	24.50 (7.77)		6.00 (1.41)		4.50 (2.12)	
Civil status		0.2694 (0.049) *		1.826 (0.151)		0.719 (0.544)
Single	33.09 (11.60)		7.00 (2.09)		6.63 (2.01)	
Married/in a relationship	25.21 (12.56)		8.34 (1.47)		7.03 (2.22)	
Separated/divorced	32.00 (13.92)		7.80 (1.83)		7.19 (2.29)	
Widower	19.83 (7.88)		7.66 (1.75)		5.83 (0.75)	
		***r* (*p*)**		***r* (*p*)**		***r* (*p*)**
Age		−0.229 (0.048) *		0.228 (0.049) *		0.015 (0.901)
AUDIT		0.455 (<0.001) *		0.062 (0.604)		0.259 (0.028) *
Abstinence duration (days)		−0.132 (0.269)		0.087 (0.467)		0.010 (0.931)
STAI-trait		0.367 (0.002) *		−0.069 (0.566)		0.074 (0.537)
Stroop Task		−0.119 (0.320)		0.076 (0.526)		0.071 (0.555)
MACS		NA		−0.009 (0.940)		0.128 (0.283)

*M* = mean; *SD* = standard deviation; *t* = independent *t*-test; *F* = analyses of variance (ANOVA); *r* = Pearson correlation; AUDIT = Alcohol Use Disorder Identification Test; MACS = Multidimensional Alcohol Craving Scale; MACS-VR = Multidimensional Alcohol Craving Scale—Virtual Reality; PR = perceived realism; STAI-trait = trait subscale of State-Trait Anxiety Inventory; NA = not applicable; * *p* < 0.05.

**Table 3:** Relationships between PR of environments, PR of beverages and MACS-VR.

	*M* (*SD*)	*r* (*p*)		
Characteristics	Total Sample	MACS-VR	PR of Environments	PR of Beverages
MACS-VR	27.87 (12.96)	NA	0.149 (0.213)	0.362 (0.002) *
PR of environments	7.90 (1.73)	0.149 (0.213)	NA	0.578 (<0.001) *
PR of beverages	6.93 (2.11)	0.362 (0.002) *	0.578 (<0.001) *	NA

*M* = mean; *SD* = standard deviation; *r* = Pearson correlation; MACS-VR = Multidimensional Alcohol Craving Scale—Virtual Reality; PR = perceived realism; NA = not applicable; * *p* < 0.05.

**Table 4:** Multiple regression analyses with intensity of alcohol craving experienced during VR exposure, PR of environments and PR of alcoholic beverages as the dependent variables.

**MACS-VR**	**B**	**SE B**	***β***	***t***	***p* Value**
Civil status	−0.010	1.702	−0.001	−0.006	0.996
Age	−0.124	0.171	−0.083	−0.723	0.472
AUDIT	0.344	0.160	0.273	2.154	0.035 *
STAI-trait	0.192	0.135	0.176	1.426	0.159
PR of beverages	1.741	0.655	0.285	2.660	0.010 *
**PR of Environments**	**B**	**SE B**	***β***	***t***	***p* Value**
Education level	−0.336	0.129	−0.241	−2.603	0.011 *
Age	0.036	0.018	0.184	1.999	0.049 *
PR of beverages	0.444	0.075	0.544	5.933	<0.001 *
**PR of Alcoholic Beverages**	**B**	**SE B**	***β***	***t***	***p* Value**
MACS-VR	0.037	0.017	0.227	2.174	0.033 *
AUDIT	0.025	0.021	0.122	1.185	0.240
PR of environments	0.657	0.114	0.537	5.772	<0.001 *

AUDIT = Alcohol Use Disorder Identification Test; MACS-VR = Multidimensional Alcohol Craving Scale—Virtual Reality; PR = Perceived Realism; STAI-trait = State-Trait Anxiety Inventory; NA = not applicable; * *p* < 0.05.
